# Current State-of-the-Art and Perspectives in the Design and Application of Vitrimeric Systems

**DOI:** 10.3390/molecules30030569

**Published:** 2025-01-27

**Authors:** Diego Pugliese, Giulio Malucelli

**Affiliations:** 1 Istituto Nazionale di Ricerca Metrologica (INRiM), Strada delle Cacce 91, 10135 Torino, Italy; 2 Department of Applied Science and Technology, Politecnico di Torino, Viale Teresa Michel 5, 15121 Alessandria, Italy; giulio.malucelli@polito.it

**Keywords:** vitrimers, thermoplastics, thermosets, covalent adaptive networks, dynamic exchangeable linkages, recyclability, reshapeability, self-healing

## Abstract

To fulfill the current circular economy concept, the academic and industrial communities are devoting significant efforts to plastic materials’ end-of-life. Unlike thermoplastics, which are easy to recover and re-valorize, recycling thermosets is still difficult and challenging. Conversely, because of their network structure, thermosetting polymer systems exhibit peculiar features that make these materials preferable for several applications where high mechanical properties, chemical inertness, and thermal stability, among others, are demanded. In this view, vitrimers have quite recently attracted the attention of the scientific community, as they can form dynamic covalent adaptive networks that provide the properties typical of thermosets while keeping the possibility of being processed (and, therefore, mechanically recycled) beyond a certain temperature. This review aims to provide an overview of vitrimers, elucidating their most recent advances and applications and posing some perspectives for the forthcoming years.

## 1. Introduction

For many years, the polymer science and technology sector has been characterized by a distinct separation between thermoplastics and thermosets. In fact, the former exhibit a different behavior with respect to the latter, as far as their structure, thermal, mechanical, and processing characteristics are considered. Thermoplastics, indeed, can be subjected to repeated heating–forming–cooling cycles, as their polymer chains do not exhibit any crosslinking degree, i.e., any interconnection among them. Conversely, thermosetting polymers undergo the forming process during the first heating up, as they give rise to 3D insoluble and unmeltable networks [1,2,3]. If, on the one hand, the so-obtained networks are responsible for a higher thermo-mechanical behavior possessed by thermosets compared to thermoplastics, on the other hand, they usually account for difficult recyclability and reprocessability.

This large gap was partially filled with the discovery of vitrimers, i.e., a sort of intermediate polymer class, in 2011, by Montarnal and co-workers [4]. In particular, they verified the possibility of synthesizing specific polyester epoxy resins embedding a zinc transesterification catalyst suitable for the on-demand (i.e., upon heating) interchain transesterification reactions: as a consequence of the dynamic rearrangement of the polymer networks, the polyester epoxy resins were capable of reflowing under the application of an external stress, hence becoming reshapeable and recyclable.

Thus, the key concept in vitrimers relies on the presence of dynamic covalent bonds in their polymer networks, which accounts for their reshapeability, reprocessing, and even recyclability upon heating over a specific (transition) temperature. Conversely, vitrimers maintain the thermal and mechanical features of typical thermosetting systems below this temperature. In addition, this transition from thermosetting to thermoplastic behavior allows for defining the structure of the vitrimers as that of a covalent adaptive network (CAN) [5,6,7,8,9].

In particular, the presence of reversible chemical linkages provides the CAN with the possibility of restructuring itself according to such environmental stimuli as pressure and temperature without requiring important changes in the chemical composition of the vitrimer system. Further, the response from the vitrimeric materials to the applied external stimuli improves the material’s end-of-life, hence suggesting their potential use in advanced sectors that comprise electronics, materials science, and biomedical engineering, among others, where the exploitability of long-lasting structures and components is currently very demanding [10,11,12,13,14].

**Figure 1:**
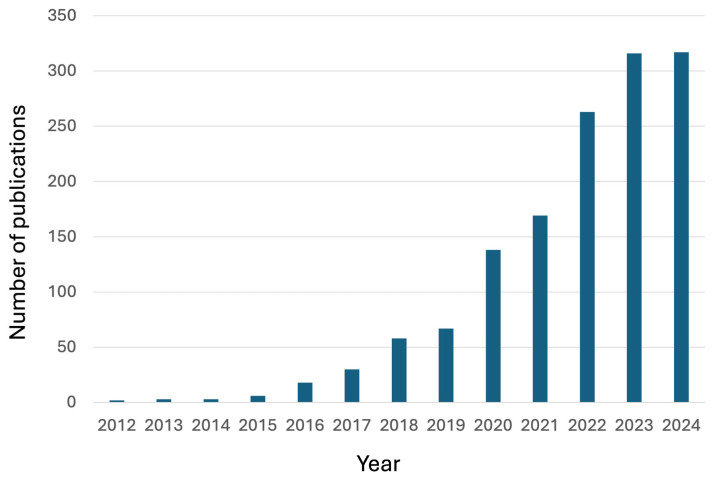
Publications (from 2012 to 2024) in peer-reviewed journals dealing with “vitrimers” (data collected from the Web of Science™ database, https://www.webofscience.com/, accessed on 1 December 2024).

Since their discovery, the attention paid to vitrimers has significantly increased, as evidenced by the large and remarkably growing number of publications that appeared in the scientific literature (Figure 1): indeed, new types of dynamic covalent bonds and new vitrimerization chemistries have successfully been designed.

The present review work is aimed at illustrating the vitrimeric concept, the chemistries behind vitrimerization, and some recent applications of vitrimers in advanced sectors, highlighting the potential of this up-to-date class of polymers for designing cutting-edge innovative materials.

## 2. Covalent Adaptive Networks at a Glance

**Figure 2:**
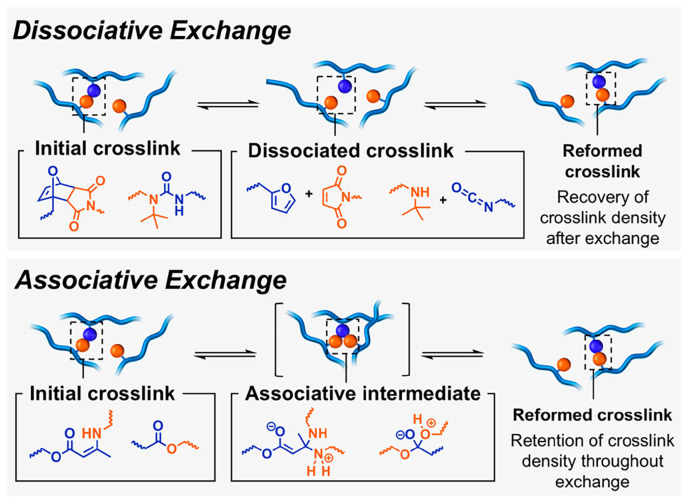
Scheme of dissociative and associative bond exchange pathways for CANs. Reprinted with permission from [26]. Copyright American Chemical Society, 2019.

**Figure 3:**
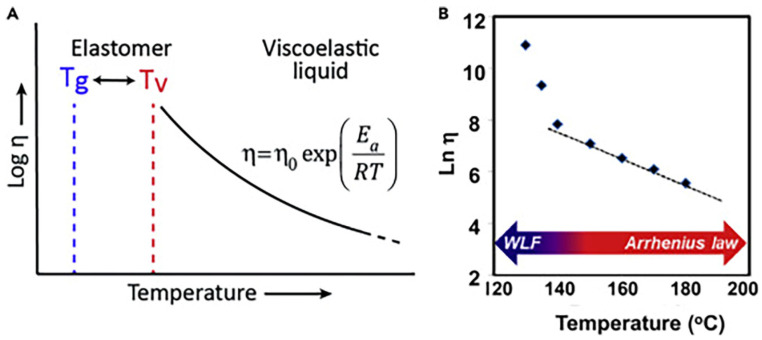
Temperature dependence of viscosity in CANs: (**A**) When *T_v_* > *T_g_*, the viscosity follows an Arrhenius law in the proximity of *T_v_*. (**B**) When *T_g_* > *T*_v_, the viscosity first follows the Williams–Landel–Ferry (WLF) law in the proximity of *T_g_* and then an Arrhenius law at adequately higher temperatures. Reprinted with permission from [28]. Copyright Elsevier, 2019.

Two temperatures (*T_g_* and *T_v_*, the “traditional” glass transition temperature, and the topology freezing transition temperature, respectively) and two possible exchange pathways (i.e., associative and dissociative, Figure 2) are the key characteristics of covalent adaptive networks (CANs). In particular, when a CAN approaches *T_v_*, its network topology reorganizes thanks to the occurrence of fast exchange reactions, which allow the material to shift from a viscoelastic solid to a viscoelastic liquid state (Figure 3). In these conditions, the CAN viscosity achieves values of about 10^12^ Pa·s, and the reversible reactions involving the dynamic bonds start, hence enabling the polymer network to undertake structural changes [15]. Beyond *T_v_*, the CAN viscosity is observed to obey the Arrhenius law, which is determined by the kinetics associated with the reversible cleavage and reforming of bonds. It is noteworthy that, although *T_v_* is usually higher than *T_g_*, bond reversibility may occur below *T_g_* in some cases.

Then, according to the associative pathway, CANs undergo bond exchanges without significant modifications in the crosslinking density during the break and reforming of chemical bonds, thus not affecting the macromolecular structure of the network. Conversely, the dissociative pathway is usually responsible for a significant decrease in the crosslinking density of the polymer networks as a consequence of the loss in their connectivity [16]. Therefore, these findings have suggested classifying associative CANs as the only types of vitrimers [12,17,18,19]. At variance, the scientific literature reports several examples of dissociative CANs that demonstrate an Arrhenius behavior over their reprocessing temperatures and have been described as “vitrimer-like”, showing subtle or negligible differences in features with vitrimeric associative CANs. This outcome casts doubt on the value of classifying these materials based on the crosslink exchange mechanism [20,21,22,23,24,25,26,27].

The main functional groups and the reactions that take place during the vitrimerization processes will be summarized in the following subsections.

### 2.1. Disulfide Bonds

**Figure 4:**
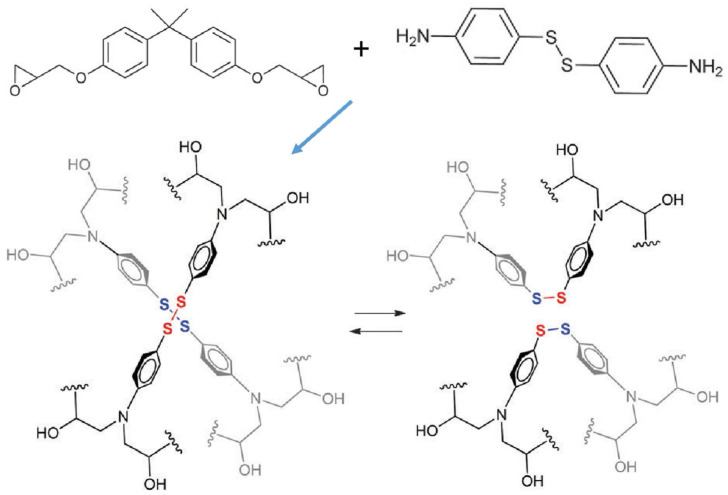
Synthesis of epoxy vitrimers based on disulfide bonds and their exchangeable reaction. Reprinted with permission from [37]. Copyright Elsevier, 2021.

Disulfide bonds are relatively weak covalent bonds, and their synthesis, usually performed at low temperatures, is strictly dependent on both temperature and pH values, which can remarkably affect the thermal stability of the resulting disulfide-containing materials [29]. Disulfide bonds are able to provide the CANs with bond exchangeability at low temperatures while maintaining acceptable bond strengths. Disulfide bonds are generally exploited for the synthesis of epoxy vitrimers [30]; to this aim, epoxy resins are cured with disulfide-containing compounds, such as aromatic diamines (Figure 4) [31,32], acids or anhydrides [33,34], and thiols [35,36], in the presence of appropriate catalysts.

### 2.2. Boronic Esters

**Figure 5:**
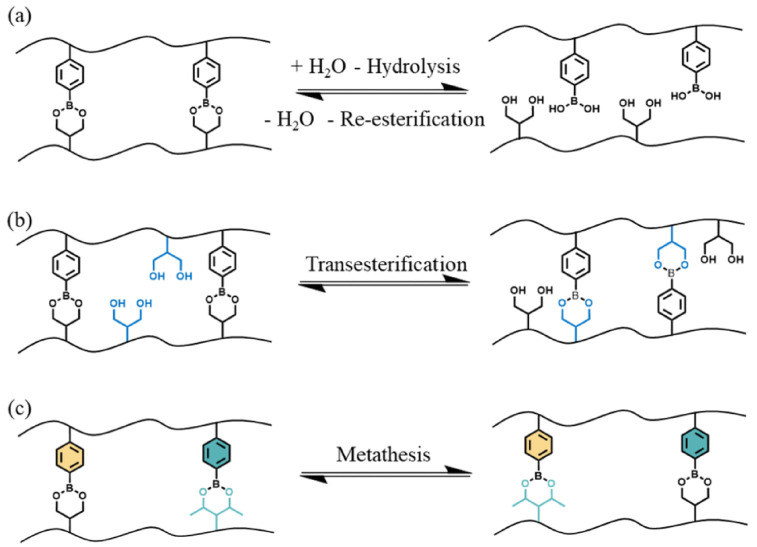
Three possible exchange mechanisms of boronic esters: (**a**) hydrolysis/re-esterification, (**b**) transesterification, and (**c**) metathesis. Black lines represent polymer chains of the crosslinked network. Reprinted from [40] under CC BY 3.0 License.

Boronic esters are synthesized through the reaction of boronic acids and alcohols, hence resulting in the formation of a product bearing a boron atom simultaneously linked to a carbon atom and an alkoxy group. It is noteworthy that the reaction is an equilibrium, where the forward reaction is thermodynamically favored when the boronic ester product is not soluble in the reaction medium [38]. Boronic esters gathered great attention thanks to their high thermal stability, limited sensitivity to oxygen, easily tunable dynamic characteristics (in the absence of a catalyst), and compatibility with several functional groups [39]. Boronic ester-based vitrimers can exploit three main dynamic exchange mechanisms, namely: hydrolysis and re-esterification, transesterification with a diol, and metathesis with another boronic ester (Figure 5).

These findings are mainly due to the electron-deficient features of boron, which can interact with nucleophilic species such as water and alcohols. In addition, the dynamic exchange reactions that involve boronic esters are strictly associated with temperature and pH. In particular, low pH values favor the hydrolysis/re-esterification mechanism, thus leading to the formation of boronic acid and the related alcohol.

Very recently, Fang and co-workers exploited a one-pot reaction for synthesizing a series of boronic ester vitrimers based on poly(butyl acrylate) [41]. To generate intermolecular boron–nitrogen (B–N) coordination, the authors copolymerized dimethylaminoethyl methacrylate in the polymer backbone; this way, the B–N coordination accounted for a quick break and recombination of bonds during stretching, also highlighting fast stress relaxation rates and low activation energies.

### 2.3. Amidic and Iminic Linkages

Iminic linkages (also known as Schiff bases) can be obtained through the reaction of a carbonyl compound (e.g., an aldehyde) with an amine; the reaction is reversible and controlled by the system’s thermodynamics [42]. Reacting vanillin with an amine crosslinker (namely, cystamine) accounted for the obtainment of poly(amide–imine) vitrimers that showed self-healing features and chemical recyclability (i.e., through hydrolysis carried out in acidic conditions) [43]. Further, Liang and co-workers designed a green approach to synthesize a series of partially bio-based, malleable, recyclable, and mechanically performing poly(amide–imine) vitrimers via bulk polymerization [44]. The obtained vitrimers, because of the strong hydrogen bonding promoted by the amidic groups and, therefore, the formation of a more interconnected network, exhibited improved creep resistance, thermal stability, and mechanical features with respect to the counterparts embedding imine groups only.

### 2.4. Diels–Alder-Based Vitrimers

**Figure 6:**
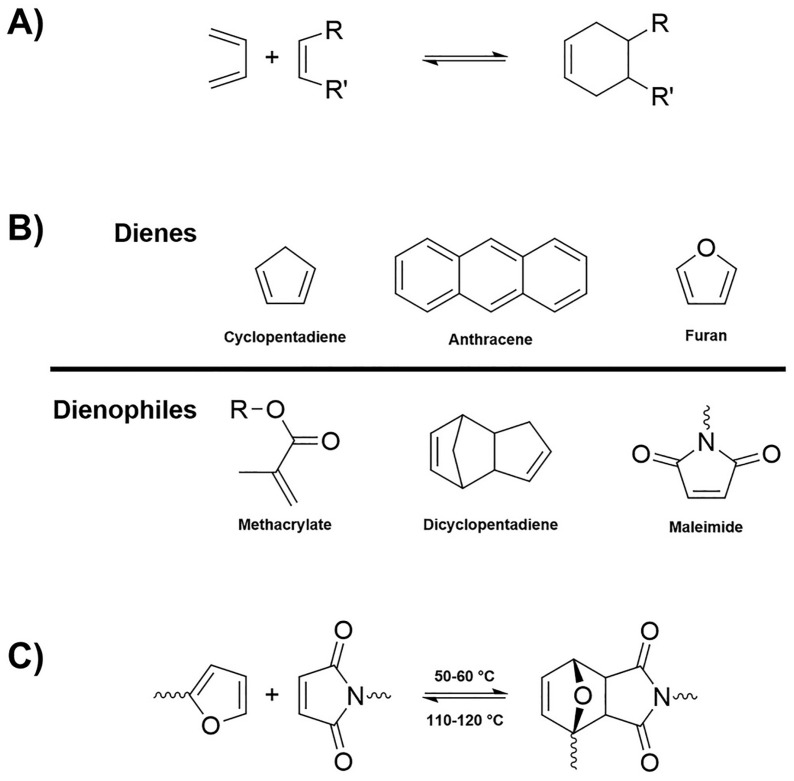
Reversible DA linkages: (**A**) general DA mechanism, (**B**) some examples of dienes and dienophiles, and (**C**) DA and retro-DA mechanism of the furan-maleimide adduct. Reprinted from [45] under CC BY 4.0 License.

Diels–Alder (DA) reactions can be successfully exploited for the synthesis of vitrimers based on dissociative bond exchange pathways. DA linkages are thermally reversible and show all the advantages related to the adopted mild reaction conditions, the possibility of avoiding the use of catalysts, and the limited formation of by-products. Quite frequently, it is possible to design vitrimeric systems based on DA reversible linkages as schematized in Figure 6.

## 3. Current Applications of Vitrimers

The self-healing, shape memory, reprocessing, and recycling abilities of vitrimers, arising from the presence of covalent and reversible dynamic bonds, make them promising materials for a variety of advanced applications, which will be detailed in the following subsections.

### 3.1. Self-Healable Materials

Self-healing in vitrimeric networks has been attributed to the existence of dynamic covalent bonds, which lead to dynamic chain-exchange reactions and topology rearrangements under external stimuli. Jiang and co-authors investigated the self-healing properties of polyurea vitrimers by using hindered urea bonds as dynamic crosslinks [46]. Upon heating, the rate of breaking and reforming of hindered urea bonds markedly increased. When the temperature was further raised to 120 °C, the exchange rate was sufficiently high to activate the polymer chains’ mobility and completely repair the scratches made on the sample. In addition, the repaired film exhibited strong mechanical properties, being stretchable up to 200% strain without tearing, as well as lifting a 0.5 kg weight without breaking at the healed part.

Another example of self-healing vitrimers using a dynamic polymer–polymer (P–P) interaction approach was reported by Wang and co-workers [47], who exploited the formation of boronic ester linkages in either poly(styrene) (PS) or poly(methyl methacrylate) (PMMA). The resulting vitrimers showed superior mechanical performances (i.e., tensile strength and Young’s modulus values of 65 MPa, and 2.1 GPa, respectively) compared to their small molecular crosslinked counterparts and corresponding thermoplastics. Upon heating, the material highlighted a fast self-repair behavior along with malleability and recyclability due to the well-dispersed dynamic covalent linkages among polymer chains.

Ying and co-workers incorporated 1-(tert-butyl)-1-ethylurea (TBEU) moieties into a crosslinkable poly(urethane-urea) to obtain a self-healing and catalyst-free vitrimeric material [48]. The TBEU exhibited a high binding constant (*K_eq_*) for reserving the strong bonding and a large dissociation constant (*k*_-1_) for high-efficiency dynamic bond exchange under mild conditions.

Kim et al. investigated the self-healing properties of a biomass-derived polyurethane (PU) vitrimer, which completely healed within 1 h at 160 °C and 30 MPa pressure [49].

Vidal and co-workers used Lewis pairs (LPs) (i.e., electron-poor Lewis acids (LAs) and electron-rich Lewis bases (LBs)) as novel dynamic crosslinkers [50]. This way, transient polymer networks were formed between LA-containing PS- and LB-terminated telechelic polydimethylsiloxane. The mechanical properties of the transient polymer network could be tuned over a wide range by choosing a suitable LP. Interestingly, due to the reversible LPs reactions, two edge-touching pieces of the polymer networks could spontaneously rejoin at room temperature within around 2 weeks.

Finally, Wang and co-workers prepared a hyperbranched epoxy vitrimer by curing bis(2,3-epoxypropyl) cyclohex-4-ene-1,2-dicarboxylate with 50 wt.% of a phosphorus/silicon-containing polyethyleneimine at room temperature. The rational integration of dynamic non-covalent hydrogen bonding, π-π stacking, and covalent β-hydroxy ester bonds into the high-mobility branched units of the vitrimer network allowed achieving an outstanding room temperature self-healing efficiency up to 96.0% [51].

### 3.2. Adhesive Applications

Another facet of processing, for which vitrimers are well-suited, is the ability to weld objects together. Effectively welding different polymeric materials is of great interest in different fields, including microfluidic devices, packaging, robotics, and electronics [12].

The formation of interfacial crosslinks reinforces the P–P interface and allows for the occurrence of strong adhesion phenomena between vitrimeric objects [52]. Compared to traditional adhesives based on epoxy resins, phenol–formaldehyde resins, organosilicons, and urea–formaldehyde resins, vitrimeric adhesives exhibit such outstanding properties as high transparency, strong adhesion, excellent mechanical properties, recoverability, as well as chemical resistance [53,54].

Capelot and co-workers reported the welding of epoxy–anhydride and epoxy–acid vitrimers together through heat pressing [55]. They varied the temperature, time, and catalyst concentration in the vitrimers and showed that enhancing catalyst loading accelerated welding, while a high alcohol content facilitated the achievement of greater weld strengths.

**Figure 7:**
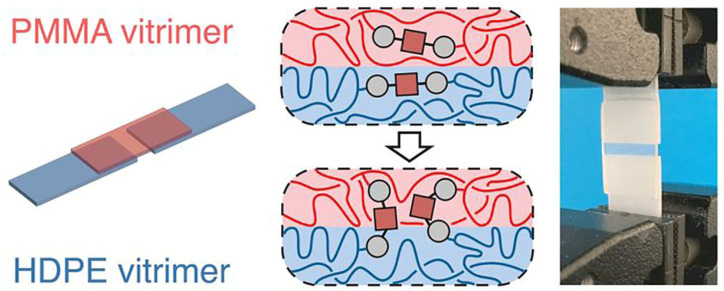
Schematic representation and photo highlighting enhanced adhesion of PMMA and HDPE vitrimers. Adapted with permission from [56]. Copyright American Association for the Advancement of Science, 2017.

In a more recent work, Röttger and co-workers demonstrated the successful welding of dioxaborolane-based vitrimers of high-density polyethylene (HDPE) and PMMA (Figure 7) [56]. After having been pressed together at 190 °C at 11 kPa of pressure for 20 min, the adhesion between the two materials was so strong that mechanical failure occurred in the PMMA bulk and not at the joint. This ability to easily form and reform bonds provides interesting implications for repairing damage in optical and electronic devices.

Zhang and co-workers demonstrated a fully bio-based epoxy vitrimer system (Se-EP/Oz-L) with high lignin content as a recoverable adhesive [57]. The latter, unlike the usual disposable counterparts, provided the aluminum parts with the ability to reversibly detach and re-bond on demand due to the robust transesterification exchange reactions taking place at elevated temperatures.

Another vitrimeric adhesive based on dynamic poly(thiourethane) (PTU) was prepared by Cui et al. by introducing exchangeable thiocarbamate bonds [58]. The obtained transparent PTU adhesive exhibited good water resistance, reprocessability, and outstanding bonding performance for different types of substrates including glass, metal, and wood.

Then, a series of epoxy-based vitrimer materials were prepared by Surós et al. from a diglycidyl ether of bisphenol A epoxy resin, trimethylolpropane, succinic anhydride as curing agent, and 1-methylimidazole as catalyst [59]. Different methodologies of re-adhesion with different bond thicknesses were explored, namely: re-adhesion after joint breaking, re-adhesion after joint dismantling, and adhesion after curing separately the formulation on the adherend and by self-welding, reaching values of re-adhesion up to 96% with respect to the original lap shear stress.

### 3.3. Recyclability/Reprocessing Applications

**Figure 8:**
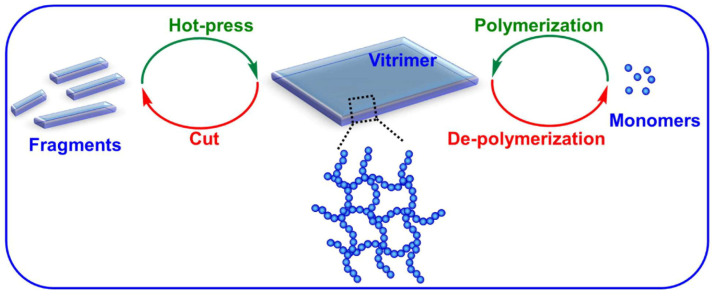
Schematic illustration of the recycling mechanisms of vitrimers. Reproduced with permission from [12]. Copyright Elsevier, 2021.

Vitrimers are potentially able to function as a type of recyclable thermosets thanks to their peculiar chemistry. The recycling of vitrimers is typically carried out through two main mechanisms, namely hot-press and solvent dissolution (Figure 8) [12].

In most cases, vitrimers can be reprocessed by hot-pressing their fragments or debris into a new robust integrated film due to their dynamic covalent bonds [18]. Taplan and co-workers also reprocessed imine-based vitrimers using the extrusion process [60]; the reformed vitrimeric specimen contained a continuous network architecture analogous to the pristine sample and showed a comparable mechanical response.

Catalyst-free, fully bio-based vitrimers featured by high mechanical strength and re-cyclability were synthesized by Li and co-workers starting from tannic acid, epoxidized vegetable oils, and maleic anhydride [61]. The vitrimers showed a tensile strength ranging from 21.9 to 52.4 MPa, and they were able to withstand at least 3 rounds of mechanical recycling while still retaining a tensile strength higher than 10 MPa. The unique branched poly-ring structure of tannic acid was responsible for the perfect combination of robust mechanical properties and good reprocessability, allowing for the formation of a hyperbranched network that exhibited both high mechanical strength and chain mobility.

The second vitrimer reprocessing system is solvent depolymerization of the polymer network into its constituent monomers or oligomers. After the evaporation of the solvent, these extracted components can be re-polymerized and a new vitrimeric network originates. As a representative example, Jing and Evans designed a poly(ethylene oxide) (PEO)-based network containing dynamic boronic ester bonds for ion transport and electrolyte applications [62]. The mechanical properties and electrical conductivity of the vitrimer were correlated to the concentration of (trifluoromethanesulfonyl)imide (LiTFSI). This polymer could then be dissolved in water, and recycled back to its original monomers, which could be re-polymerized to generate a new vitrimer with comparable conductivity.

Another example was reported by Zhou and co-workers, who used 4-aminophenyl disulfide as a multifunctional amine crosslinker in an amine-epoxy system to induce recyclability [63]. In this synthesized network, both heating and pressing could activate the disulfide exchange reaction, resulting in the rearrangement of the network topology. Both hot-pressing and solution processing played a key role in reprocessing, and after recycling and reprocessing, mechanical properties comparable to those exhibited by the original sample were found.

Other examples of a solution processing mechanism include the boroxine-based vitrimers reported by Ogden and Guan [64] and the poly(diketoenamine) vitrimers described by Christensen and co-workers [65].

### 3.4. Shape Memory Materials

Shape memory polymers (SMPs) are a special class of crosslinked elastomers that exhibit programmable shape-morphing behavior in response to external stimuli. Among other applications, they have enormous potential for use in biomedical devices, smart actuators, robotics, electronic devices, and automation equipment [12].

Traditional SMPs show a dual deformation behavior. At a temperature beyond *T_g_*, the polymer chain segment is activated to permit the deformation under the application of an external force, lowering the entropy of the system. After cooling, the distorted shape will momentarily be fixed. The deformed polymer can regain its original shape when heated above *T_g_* due to the entropic nature of the chain conformation change. This dual shape-morphing behavior directed by entropy changes is known as polymer elasticity. Plasticity, which is typically in opposition to elasticity, is another significant polymer behavior consisting of the ability of polymers with dynamic exchangeable bonds to permanently change their shape in response to external forces [12].

Traditional SMPs exhibit only a single shape-changing cycle, thus they are unable to meet the growing demand for geometrically complex multifunctional devices. In contrast, vitrimers, combining both elasticity and plasticity, could be programmed with multiple shape memory effects. This makes it possible for the material to continuously change into a variety of intricate and geometrically complex 3D structures, which may be helpful in aerospace and soft robotics applications. The vitrimer is initially programmed at a temperature higher than *T_v_*, whereby the dynamic covalent bond is sufficiently activated to reorganize the network topology under an external load. Upon cooling, a new permanent shape is acquired without entropy changes during topology rearrangement; as a result, this new permanent shape is irreversible and will remain intact upon temperature changes in the absence of applied external stresses [66].

In this context, Zhao and co-workers demonstrated a paradigm for adding plasticity to an elastic polymer network by preparing a structure bearing ester crosslinkers that exhibited both elasticity and plasticity under different triggering temperatures using poly(caprolactone)-diacrylate and a tetrathiol crosslinker [67]. The cumulative nature of the plasticity in this system made it possible to fabricate parts with intricate 3D structures that were otherwise be thought to be impossible.

By adding an interchangeable transcarbamoylation reaction, the same group also altered a traditional shape-changeable PU thermoset to demonstrate its reworkability [68]. The modified PU thermoset contained both thermal phase transition and dynamic covalent motifs and could undergo multi-type shape reshuffling.

**Figure 9:**
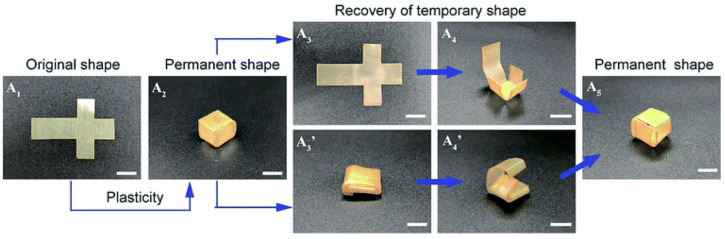
Visual demonstration of thermadapt shape memory behavior of an EPSi specimen (scale bar: 10 mm). Legend: A_1_ = original plane shape of the specimen; A_2_ and A_5_ = permanent shape of the specimen; A_3_ = plane unfolded temporary shape; A_3_’ = folded temporary shape; A_4_ = intermediate shape recovery step between A_3_ and A_5_; A_4_’ = intermediate shape recovery step between A_3_’ and A_5_. Adapted with permission from [69]. Copyright Royal Society of Chemistry, 2019.

Robust thermadapt SMPs featuring high *T_g_* are also highly desirable in high-performing smart devices with self-folding and self-deployable properties. Within this framework, Ding and colleagues reported a novel thermadaptable shape memory polymer (EPSi) based on dynamic silyl ether linkages (Figure 9) [69]. EPSi demonstrated a high *T_g_* that could be adjusted from about 118 to 156 °C without sacrificing its mechanical strength. Interestingly, EPSi showed outstanding thermadapt self-folding properties, with a shape fixity ratio ranging from 97.1 to 98.9%, a shape recovery ratio between 95.6 and 99.8%, and a good shape retention ratio (from 80.5 to 86.3%).

Yuan and co-workers fabricated sequence-rearranged reconfigurable co-crystalline networks, which demonstrated significant promise for use in biomedical devices [70]. Due to the dynamic transesterification nature, the resulting network showed a tunable melting point, relatively high crystallinity, and excellent shape reconfigurability properties at body temperature.

Asempour et al. reported a catalyst-free vinylogous urethane vitrimeric rubber based on β-myrcene and β-ketoester functional (acetoacetoxy)ethyl methacrylate copolymers [71]. The vitrimers demonstrated a shape memory effect with high shape fixity and quick shape recovery (<1 min) when heated above their *T_g_*. Moreover, the vitrimers could experience permanent shape reprogramming through the reconfiguration of dynamic bonds at about 110 °C.

### 3.5. Soft Actuators and Liquid Crystalline Elastomers

Soft actuators with pre-set shapes undergoing a defined or programmed movement in response to external stimuli (i.e., heat, light, solvent, and electricity) are highly desirable in fields like soft robotics, energy generators, motors, and fluid propellers [12]. Among various techniques, liquid crystalline elastomers (LCEs) with reversible deformation properties have garnered a lot of interest. Generally, under the application of an external load, LCE chains and mesogens experience gradual orientated aligning, and the mesogen alignment can be fixed by crosslinking. On the other hand, by selectively adjusting the liquid crystalline alignment and its crosslinking distribution, LCE actuators can undergo an order–disorder phase-transition, paving the way toward the possibility of manufacturing controllable reversible actuators [10].

Within this framework, Hanzon and co-workers engineered a novel adaptable main-chain LCE system based on thermally induced transesterification [72]. In addition to isotropic phase transition, this network exhibited unique characteristics like malleability, stress relaxation, surface welding, and recyclability.

Saed and colleagues developed a new class of LCEs by introducing the exchangeable boronic–ester reaction through a Michael-addition thiol–acrylate reaction [73]. They implemented the concept of “partial vitrimer network” material, in which the exchangeable and permanent networks are combined into a single material [74,75]. To achieve a “sweet spot” material with an ideal balance between elasticity and plasticity, the ratio of the corresponding components was rationally adjusted to tune the fraction of permanent and exchangeable parts in the network. In addition to minimizing creep, such a material system was able to preserve the network integrity.

**Figure 10:**
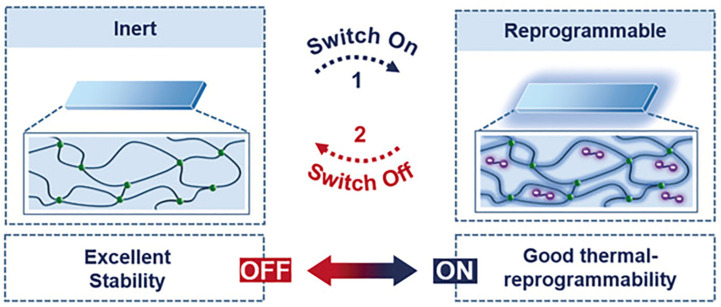
Illustration of switching on/off the thermal reprogrammability of a siloxane network through the swelling–heating method. Adapted with permission from [76]. Copyright Wiley, 2020.

Very recently, Wu and co-workers developed an effective swelling–heating method to reversibly turn on/off the thermal reprogrammability of a common siloxane LCE, fundamentally solving the conflict between the operation stability and thermal reprogrammability (Figure 10) [76]. In this system, an anionic catalyst (i.e., bis(tetramethylammonium) oligodimethyl-siloxanediolate) was used to regulate the switching functionality of the network reprogrammability by modifying the catalytic ability. Because of its high solubility, the catalyst was able to enter the network when it was immersed in a catalyst-solvent solution; this caused the siloxane chain to rearrange, which could be stopped by heating (catalyst deactivation). Interestingly, the polymer remained stable even at elevated temperatures, and the network dynamics could be continuously turned on and off by repeating the swell/heating cycle.

Trinh and co-workers fabricated a number of catalyst-free liquid crystalline epoxy vitrimer (LCEV) systems based on LCEV monomers that were cured by cationic and anionic initiators to enhance functionality [77]. The final products exhibited outstanding mechanical properties and a high thermal conductivity (~0.67 W m^–1^ K^–1^), which is three times higher than that of conventional epoxy resins (~0.2 W m^–1^ K^–1^). Additionally, the LCEVs showed excellent reprocessability with only basic heating and compression, obviating the requirement for external catalysts. Crucially, despite minor mechanical property reductions, these materials maintained their properties even after multiple reprocessing cycles.

### 3.6. Electrolyte/Battery Applications

In recent decades, vitrimers have attracted an always growing interest as a suitable alternative to traditional liquid electrolytes for battery applications, thanks to their self-healing properties and reprocessability and recyclability, which lead to an increase in the effective life span and to a decrease in the environmental issues, respectively [66].

In this context, Gu and co-workers developed a novel matrix based on soy protein isolate, which is a dynamic polymer network based on imine exchange bonds [78]. When the LiTFSI salt was added to this vitrimeric electrolyte network, the conductivity rose to 3.3 × 10^−4^ S cm^−1^ at 30 °C.

Katcharava et al. investigated a solvent and catalyst-free vitrimeric poly(ionic liquid) for the design of lithium-ion batteries featured by the following characteristics: ion conductivity, self-healing, non-flammability, reprocessability, and recyclability [79]. This vitrimeric network was synthesized using a copolymer embedding a pyrrolidinium ionic liquid moiety as the conductive component and PEO-functionalized styrene to increase the lithium-ion transfer rate. Furthermore, the vitrimeric properties were provided by the employment of boric acid in the synthesis process.

The use of healable ionogels based on vitrimer chemistry has also become an effective strategy to increase and improve the cyclic life of electrolytes. In this regard, Li et al. developed S-transalkylation polythioether networks [80]. Then, ionogels with good ionic conductivity were prepared by adding 1-ethyl-3-methylimidazolium bis(trifluoromethylsulfonyl) imide or 1-ethyl-3-methylimidazolium trifluoromethanesulfonate.

Moreover, the use of an electrolyte made of a PEO-based network containing dynamic boronic ester bonds has also been reported [62]. The concentration of LiTFSI salt revealed to be one of the key elements influencing the mechanical characteristics and ionic conductivity of this vitrimeric electrolyte. Also, this latter showed the ability to dissolve in water and turn into its primary monomers.

Lastly, vitrimer solid polymer electrolytes (V-SPEs) with dynamic imine bonds and high ionic conductivity of up to 3.25 × 10^−4^ S cm^−1^ at 60 °C were synthesized by Yang et al. for use in lithium metal batteries (LMBs) [81]. Interestingly, the imine exchange reaction endowed self-healing capability to V-SPEs at ambient temperature. The LMB full cell employing V-SPE exhibited a large initial discharge capacity of 154.7 mAh g^−1^, good C-rate capability, and long cycle lives for at least 100 charge–discharge cycles carried out at 60 °C and 0.1 C.

### 3.7. Three-Dimensional and Four-Dimensional Printing

Three-dimensional printing is a very effective technique in designing complex geometries with high accuracy and speed. However, conventional 3D objects fabricated by the layer-by-layer construction strategy generally do not show proper mechanical properties, which limits their applicability in practical engineering. The combination of 3D printing technology and vitrimer chemistry results in recyclable 3D devices with sophisticated structures and precise control over the fabrication process without sacrificing object robustness. This allows for large-scale applications in a variety of fields, such as electronic/optical materials, SMPs, and actuators.

**Figure 11:**
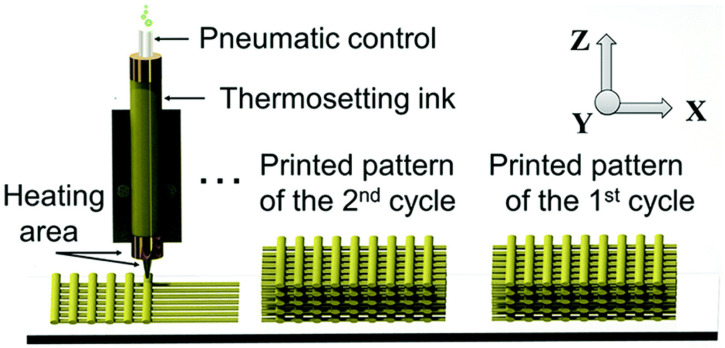
Extrusion-based 3D printer equipped with heating elements for recyclable printing of epoxy vitrimer. Adapted with permission from [82]. Copyright Royal Society of Chemistry, 2017.

In this regard, Shi and co-workers developed a 3D printing ink based on a recyclable epoxy vitrimer (Figure 11) [82]. This ink could be printed into geometrically complex parts and subsequently recycled by dissolving it in hot ethylene glycol to generate a new vitrimer ink for the following 3D printing cycle. The designed vitrimer ink could be printed four times while retaining outstanding printability.

Rossegger and co-workers developed a novel liquid transesterification catalyst (i.e., oligomeric methacrylate phosphate) with excellent solubility in a variety of acrylates, good stability in the thiol–click reaction, and the ability to be covalently bonded to the network [83]. This catalyst demonstrated outstanding catalytic activity in thiolacrylate vitrimers with enhanced mechanical properties. The prepared materials could be processed using digital light to manufacture accurate 3D soft active devices with triple-shape memory and self-repair ability.

Gao and co-workers developed a robust and reprocessable acrylate vitrimer for the photo-3D printing application via combing exchangeable β-hydroxyl esters and sacrificial hydrogen bonds based on the reaction between diacrylate prepolymer, tetrahydro–furfuryl acrylate, and acrylamide [84]. When the acrylamide content was raised up to 20 wt.%, the resulting vitrimer maintained its reprocessability while demonstrating significantly higher tensile strength (about 40 MPa) and Young’s modulus (871 MPa) compared to unmodified acrylate vitrimers. This material was used to precisely fabricate recyclable 3D objects with multiple structures by using stereolithography.

Vilanova-Pérez and co-workers have successfully synthesized two novel ultraviolet (UV)-curable vitrimeric materials using cost-effective and readily available commercial reagents [85]. Ethylene glycol phenyl ether methacrylate and poly(ethylene glycol) methyl ether methacrylate were used as reactive diluents to increase the processability and recyclability of the UV-cured final polymers. Stress relaxation tests highlighted that both materials were able to relax 63% of the initial stress in less than 10 min at 110 °C. The recyclability of the materials was also achieved, and the mechanical and thermo-mechanical properties of the recycled samples were compared to the virgin ones revealing a great recovery of the initial properties. Lastly, complex structures were printed with high resolution using the digital light processing technique, demonstrating the enormous potential of these vitrimeric materials in 3D printing.

4D printing has recently been introduced in many application fields like medicine and soft robotics as a sustainable 3D printing method for responsive polymers. Therefore, the synthesis of polymer networks containing dynamic covalent bonds can provide unique properties such as shape memory, shape recovery, self-healing, and flexibility to a 3D printed part. Thus, the use of vitrimer chemistry accounted for the development of the next-dimensional printing process [86].

In this context, Kasetaite and co-workers investigated a vanillin-based vitrimer with 3D printing capability [87]. This amorphous network exhibited outstanding self-healing properties and a good shape-recovery rate.

Deng and co-workers also studied a jigsaw puzzle-like healable triboelectric nanogenerator for self-powered wearable electronics based on an elastomeric vitrimer [35]. The vitrimer-like elastomer’s dynamic disulfide exchange reaction gave the device a number of intriguing properties, including exceptional conformability and deformability, high elasticity and stretchability, and quick recovery from scratches and breaks when stimulated by heat or intense pulse light.

## 4. Conclusions and Perspectives

Undoubtedly, polymer science and technology have greatly benefited from the discovery and development of vitrimers, thanks to the unique peculiarities of the latter. Indeed, vitrimers possess reshapeability, formability, and recyclability, among other characteristics, which make them very attractive and up-to-date materials, especially within the current and highly demanding concept of circular economy. Further, the possibility of using them as a valid alternative to standard thermosets that, very often, lack environmental sustainability, is remarkably re-drawing the boundary between thermoplastic and thermosetting polymer systems. Indeed, vitrimers can bridge the gap between these two different classes of materials.

Despite the significant advantages provided by vitrimers, there are still some limitations and challenging issues that will further push the research on this topic toward new advances and perspectives. In particular, notwithstanding the important research efforts carried out so far, the development and implementation of the overall performance of vitrimeric systems impose the exploration of novel dynamic exchange reactions suitable for the design of new, effective, and reliable materials. It is then equally important to optimize the kinetics (i.e., the activation energies) of the already well-known vitrimeric systems: this optimization requires a deep investigation of their chemistries in terms of suitable employable solvents, possible use of effective catalysts, and/or specific additives. Next, as the reprocessability of vitrimers is strictly related to the presence of dynamic linkages in the polymer networks, while the stability (and the overall thermal and mechanical behavior) mainly relies on the presence of permanent crosslinks, a balance between these two opposite characteristics needs to be achieved and possibly ameliorated/optimized. Finally, all the scientific literature on vitrimers is currently limited to lab-scale research, though it envisages the potential of vitrimers for applications in advanced industrial sectors, both as structural and functional materials. In this context, the scaling up of the production of vitrimeric systems at the industrial level is still missing and requires further efforts not only from academia but also from the industrial world (which seems very interested in the future market exploitation of these materials).

Then, to fulfill the current circular economy approach and produce more sustainable materials, it is expected that the design, development, and implementation of vitrimers will be addressed regarding the use of bio-based materials. In this view, the recent scientific literature highlights the potential of different bio-vitrimers for advanced applications [88,89,90,91,92,93]. In addition to adding value to materials of natural origin, the use of bio-based vitrimers can also be a winning strategy from an environmental perspective by even using bio-scraps or bio-byproducts from different supply chains.

Finally, an important contribution to the development and implementation of vitrimeric systems could come from their simulation and modeling: in this context, the recent scientific literature reports some interesting papers that demonstrate the possibility of designing interesting vitrimers through molecular dynamics and computational simulations [19,94,95,96,97,98]. Undoubtedly, all these efforts will help researchers toward the simplification of the design of new vitrimeric systems, reducing the time required for the experimental activities, and further strengthening the knowledge of these intriguing materials.

We can expect, in the forthcoming years, a further step forward in the development of vitrimers, which will certainly broaden their application possibilities, improving or even widening their current potential.

## Data Availability

No new data were created or analyzed in this study. Data sharing is not applicable to this article.
